# Superior In Vitro Stimulation of Human CD8^+^ T-Cells by Whole Virus versus Split Virus Influenza Vaccines

**DOI:** 10.1371/journal.pone.0103392

**Published:** 2014-07-29

**Authors:** Benedict R. Halbroth, Alexander Heil, Eva Distler, Martin Dass, Eva M. Wagner, Bodo Plachter, Hans Christian Probst, Dennis Strand, Udo F. Hartwig, Anita Karner, Gerald Aichinger, Otfried Kistner, Katharina Landfester, Wolfgang Herr

**Affiliations:** 1 Department of Medicine III – University Medical Center of Johannes Gutenberg-University, Mainz, Germany; 2 Max-Planck-Institute for Polymer Research, Mainz, Germany; 3 Institute of Virology – University Medical Center of Johannes Gutenberg-University, Mainz, Germany; 4 Institute of Immunology – University Medical Center of Johannes Gutenberg-University, Mainz, Germany; 5 Department of Medicine I – University Medical Center of Johannes Gutenberg-University, Mainz, Germany; 6 Baxter, Vienna, Austria; 7 Department of Medicine III – University Medical Center of Regensburg, Regensburg, Germany; Deakin University, Australia

## Abstract

Pandemic and seasonal influenza viruses cause considerable morbidity and mortality in the general human population. Protection from severe disease may result from vaccines that activate antigen-presenting DC for effective stimulation of influenza-specific memory T cells. Special attention is paid to vaccine-induced CD8^+^ T-cell responses, because they are mainly directed against conserved internal influenza proteins thereby presumably mediating cross-protection against circulating seasonal as well as emerging pandemic virus strains. Our study showed that influenza whole virus vaccines of major seasonal A and B strains activated DC more efficiently than those of pandemic swine-origin H1N1 and pandemic-like avian H5N1 strains. In contrast, influenza split virus vaccines had a low ability to activate DC, regardless which strain was investigated. We also observed that whole virus vaccines stimulated virus-specific CD8^+^ memory T cells much stronger compared to split virus counterparts, whereas both vaccine formats activated CD4^+^ Th cell responses similarly. Moreover, our data showed that whole virus vaccine material is delivered into the cytosolic pathway of DC for effective activation of virus-specific CD8^+^ T cells. We conclude that vaccines against seasonal and pandemic (-like) influenza strains that aim to stimulate cross-reacting CD8^+^ T cells should include whole virus rather than split virus formulations.

## Introduction

Seasonal influenza A and B viruses cause recurrent epidemics typically during the cold months, resulting in hundreds of thousands of deaths every year. In addition, the swine-origin H1N1 virus, which has provoked the latest influenza pandemic in 2009, and the pandemic-like H5N1 virus, which was recently reported to require as few as 5 amino acid substitutions to become airborne transmissible between ferrets, create common fears of a pandemic with a highly pathogenic influenza virus [1]–[3]. Strategies to overcome this threat include the development of influenza vaccines designed to elicit protective immunity against heterologous influenza virus strains [4], [5]. Common seasonal influenza vaccines of subunit formulation efficiently generate virus-specific B-cell responses, providing protection in a considerable proportion of vaccines. However, due to the high mutation frequency of influenza virus surface proteins, the vaccination has to be refreshed every year with the adapted vaccine formulation [6]. Vaccines capable of stimulating T-cell immunity to highly conserved internal virus proteins could overcome this limitation by inducing heterosubtypic immunity including potential pandemic viruses [4], [7]. Mouse models have shown that influenza-specific CD8^+^ CTL eliminate influenza virus-infected cells very rapidly and reduce the severity and mortality of influenza disease [8], [9]. Additionally, whole virus influenza vaccines have been found to stimulate protective immunity in murine challenge models by inducing influenza-specific CD8^+^ CTL and CD4^+^ Th cells as well as neutralizing antibodies [5], [10]. Thus, there is accumulating interest in whole virus vaccination strategies that elicit influenza-specific CD8^+^ CTL responses in humans and the question if such responses are directed against seasonal and recently circulating pandemic (-like) influenza strains.

The current study directly compared influenza whole virus and split virus vaccine formulations prepared from the same individual virus stocks for the ability to stimulate influenza-specific CD4^+^ and CD8^+^ memory T cells *in vitro*. Vaccines were derived from major seasonal influenza A and B strains as well as from pandemic swine-origin H1N1 and pandemic-like avian H5N1 strains, respectively. Due to the double-inactivated non-infectious nature of the vaccines, CD8^+^ T-cell stimulation required that viral antigens present in the vaccines were cross-presented onto MHC class I molecules in APC, as has been successfully demonstrated for mature DC [11]. The study presented herein demonstrates that whole virus vaccines are clearly superior to split virus counterpart formulations in terms of their ability to mature DC and to stimulate heterosubtypic memory CD8^+^ T cells that are cross-reactive against multiple seasonal and non-seasonal influenza strains.

## Materials and Methods

### Donors

Donors were randomly selected healthy volunteers who participated in this study after written informed consent in accordance with the Helsinki Protocol and after receiving permission by the local ethics committee (Ethics Committee of the Landesärztekammer Rheinland-Pfalz). Median age was 26 years (range, 22–52 yrs; n = 12).

### Influenza vaccines

Influenza vaccines were prepared from recent pandemic (-like) and seasonal virus strains and are listed in Table 1. Influenza viruses were grown in WHO-certified Vero cell cultures. Double-inactivation of virus harvest from culture supernatant was performed by formalin and ultraviolet treatment and achieved complete inactivation of virus particles with a safety margin of at least 300% [12]. Inactivated virus was purified by continuous sucrose gradient centrifugation and ultra-/diafiltration steps [13]. Seasonal whole virus vaccines were stored at −80°C. Pandemic (-like) whole virus vaccines were supplemented with the excipient polysorbate 80 and stored at +4°C [14]. Split virus formulations were generated from the same double-inactivated whole virus stocks by single Triton X-100 detergent incubation. Subsequently, they received the polysorbate 80 additive and were stored at +4°C. Influenza hemagglutinin (HA) antigen content was determined by standard single-radial-immunodiffusion assay. Protein composition and content of vaccine formulations were compared by semiquantitative SDS-PAGE analysis [15]. Concentration and size of RNA in vaccines were measured by standard Lab-on-a Chip electrophoretic assay in an Agilent Bioanalyzer 2100 unit. Briefly, RNA was extracted from 500 µl of vaccine samples diluted in TBS to 30 µg/ml HA [16]. RNA was labeled with intercalating dye and separated over a voltage gradient at +40°C. RNA strands were detected by laser-induced fluorescence and the recorded data were translated into gel-like images (bands) and electropherograms (peaks).

**Table 1:** Strain-specific influenza vaccines including type, used abbreviation, and status of regulatory approval.

Epidemiology	Strain	Vaccine type	Abbreviation	Approval
pandemic (-like)	A/H1N1/California/07/2009	whole virus	CF-w	licensed
	A/H5N1/Indonesia/05/2005	whole virus	ND-w	approved and used for clinical trials^a^
	A/H5N1/Vietnam/1203/2004	split virus	VN-s	experimental
		whole virus	VN-w	licensed
seasonal	A/H1N1/Brisbane/59/2007	split virus	BR-s	licensed^b^
		whole virus	BR-w	experimental
	A/H3N2/Uruguay/716/2007	split virus	UR-s	licensed^b^
		whole virus	UR-w	experimental
	B/Brisbane/60/2008	split virus	BB-s	licensed^b^
		whole virus	BB-w	experimental

a results of clinical trials used to support license of A/H5N1/Vietnam/1203/2004.

b licensed as trivalent seasonal vaccine.

Optimal results in flow cytometry and ELISpot assays were obtained with 10 µg/mL (referring to HA content) of vaccines, as determined in preceding dose-titration experiments.

### T cells and dendritic cells

PBMC were isolated from buffy coats by Ficoll centrifugation and stored in liquid nitrogen. CD4^+^ and CD8^+^ T cells were positively selected from PBMC by immuno-magnetic MicroBeads (Miltenyi Biotec, Bergisch-Gladbach, Germany). DC were generated from CD14 MicroBeads-selected monocytes and matured by exogenous cytokines [17]. Immature DC were harvested on d5, then co-incubated with influenza vaccine preparations (10 µg/mL), and then cultured for further 48 h. DC with vaccine diluting agent TBS served as negative control.

In some experiments, d5-old DC were pre-treated with irreversible proteasome inhibitor epoxomicin (10 µM; Calbiochem/Merck, Darmstadt, Germany; solvent DMSO) for 1 h, then washed and loaded for 4 h in epoxomicin (2 µM)-containing medium with whole virus UR-w (10 µg/mL), split virus UR-s (10 µg/mL) or influenza A H3N2 nucleoprotein oligopeptide mix (1 µg/mL; JPT Peptide Technologies, Berlin, Germany), respectively. For infection with live influenza virus, DC received same epoxomicin treatment but were incubated at 4×10^5^ cells for 4 h with 6.5×10^3^ hemagglutination units (4×10^5^ EID50) of Puerto Rico 8 virus (Influenza A/PR/8/24 H1N1 Allantoic Fluid; Charles River Labs, Wilmington, MA, USA). After final washing, epoxomicin-treated (or DMSO-treated) antigen-pulsed or infected DC were used in ELISpot assays.

### Flow cytometric analysis

Cells were stained with FITC-, PE-, APC- or Horizon V450-conjugated mAb (Immunotech/Beckman Coulter, Marseille, France; BD Biosciences, San Jose, CA, USA; R&D Systems, Wiesbaden, Germany; Miltenyi Biotec) and analyzed on BD FACSCanto II flow cytometer (BD Biosciences). After gating on viable cells, 10^4^ or more events were evaluated using BD FACSDiva software (BD Biosciences). Dead cells were excluded by standard 7-AAD staining (BD Biosciences). Statistical data analysis was conducted with SPSS Statistics software (IBM, New York, NY, USA).

### Cytokine measurements

IL-6, IL-12, and TNF-α secretion of DC was measured by cytometric bead array (BD Biosciences) in culture supernatants of DC upon incubation with vaccine preparations for 48 h. To measure intracellular IFN-γ production of CD4^+^ and CD8^+^ cells, immuno-magnetically purified T cell subsets were incubated with autologous vaccine-pulsed DC for 20 h. Then intracellular cytokine staining (ICS) of stimulated T cell subsets was performed using Cytofix/Cytoperm Plus Kit with BD GolgiStop, following the manufacturer's instructions (BD Biosciences). Analyses of extracellular or intracellular cytokines were performed on BD FACSCanto II flow cytometer and evaluated using BD FACSDiva and FCAP Array software (Soft Flow, Inc; both BD Biosciences). IFN-α levels in DC supernatants were measured by ELISA (PBL Interferon Source, Piscataway, NJ, USA).

### IFN-γ ELISpot assay

IFN-γ ELISpot assays were performed as previously described [18]. T cells and autologous vaccine-treated DC were seeded at 1–3×10^5^/well and 1×10^4^/well, respectively. Wells containing T cells plus DC plus TBS vaccine buffer and those with T cells only served as controls. ELISpot plates were incubated for 40 h, except for 20 h assays including proteasome-inhibited DC. Spots were counted using automated ELISpot readers ImmunoSpot Series 5 Versa (C.T.L. Europe, Bonn, Germany) and Zeiss KS ELISpot 4.9 (Carl Zeiss, Jena, Germany). Data are means ± standard deviation (SD) of duplicates or triplicates, respectively.

### Confocal Laser Scanning Microscopy (LSM)

Intracellular localization of influenza virus vaccine material was analyzed using a Zeiss LSM 510-UV confocal laser scanning microscope equipped with Zeiss LSM Image Examiner software (Version 3.2.0.115, Carl Zeiss). Briefly, d5-old immature DC were pre-treated for 4 h with epoxomicin (1 µg/mL) followed by 4 h incubation with A/H3N2-Uruguay whole virus vaccine (50 µg/mL). Pulsed cells were then washed twice in Saponin buffer and stained with mouse-anti-human influenza A H3N2-specific mAb against nucleoprotein (clone 1341, Acris, Herford, Germany) together with rabbit polyclonal antibodies against human endosomal proteins Lamp1 and Rab5 (Abcam, Cambridge, UK). Cells were washed twice and stained with fluorochrome-conjugated goat-anti-mouse and goat-anti-rabbit secondary antibodies (Molecular Probes, Gibco/Life Technologies). Isotype-matched IgG antibodies were used as controls. Nuclear co-staining was performed with Hoechst 33342 dye (Gibco/Life Technologies). Permeabilization and fixation were performed in Cytoperm/Cytofix Buffer (BD Biosciences).

### Transmission Electron Microscopy (TEM)

Transmission electron microscopy (TEM) analysis was performed with a Zeiss 912 Omega microscope (Carl Zeiss, Oberkochen, Germany) at 120 kV. A preparation protocol based on high-pressure freezing and freeze substitution was used [19]. Immature DC were seeded at 2.5×10^5^ per well in DC medium in 24-well plates containing carbon-coated sapphire discs (Wohlwend, Sennwald, Switzerland) for 4 h or overnight to allow cell adherence to discs. Afterwards, virus vaccines were added at 50 µg/mL for 2 h. In some experiments DC were pre-incubated with 1 µg/mL epoxomicin for 4 h before vaccines were added. After vaccine treatment, DC were fixed and sliced with a microtome prior to TEM analysis.

## Results

### Superior DC maturation by seasonal versus pandemic (-like) vaccines

**Figure 1:**
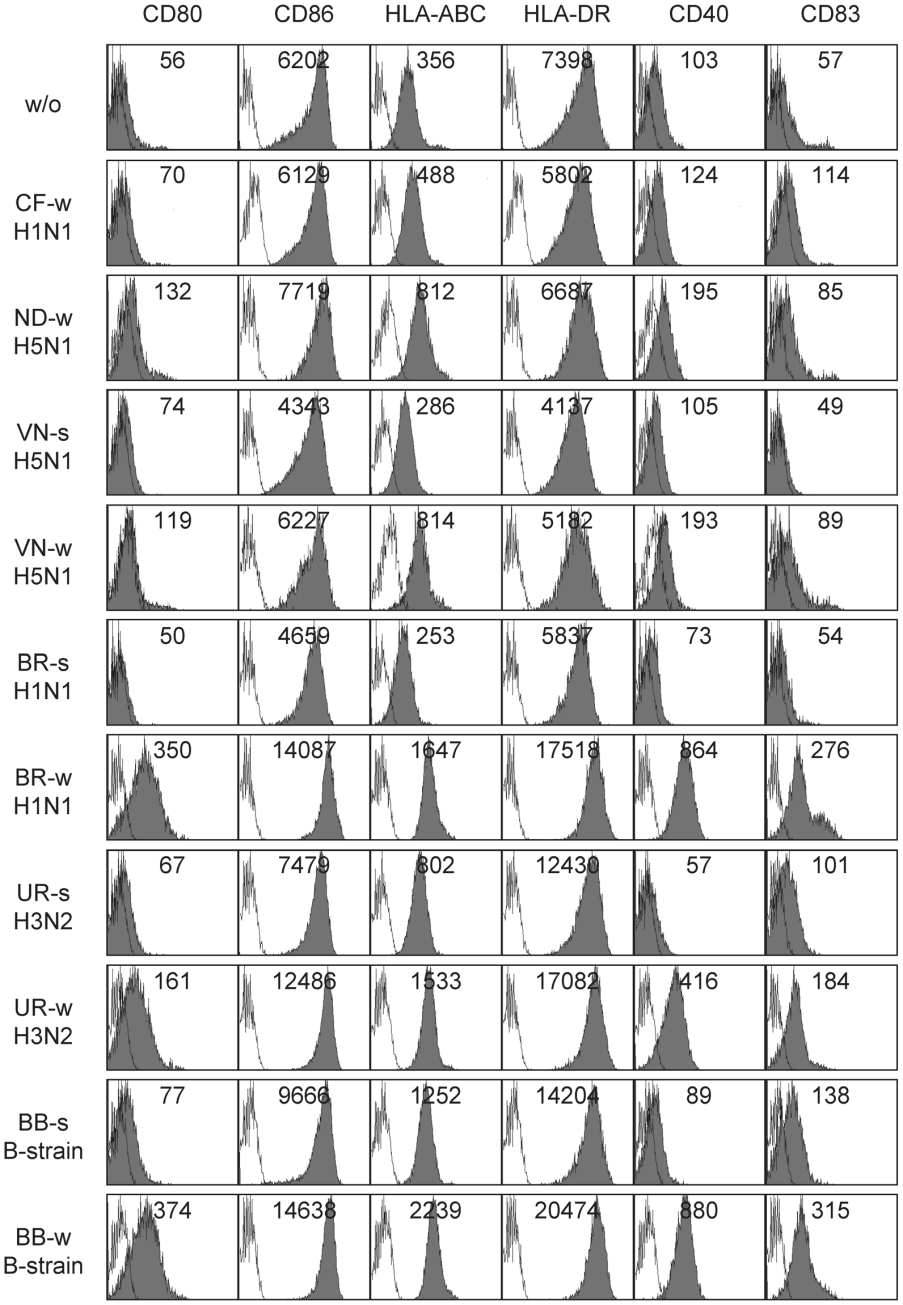
Vaccine-induced DC maturation in healthy donor HD15. Immature DC of donor HD15 were incubated for 48(-like) and seasonal influenza virus vaccines at 10 µg/mL (referring to HA content) and were subsequently analyzed by flow cytometry for expression of maturation markers on viable 7AAD-negative cells (grey histograms). Split virus formulations were unavailable from pandemic (-like) strains A/H1N1-California and A/H5N1-Indonesia. Unfilled histograms represent IgG isotype control stainings. MFI values were added to each histogram. For abbreviations of virus strains see Table 1. s, split virus; w, whole virus; w/o, without.

**Figure 2:**
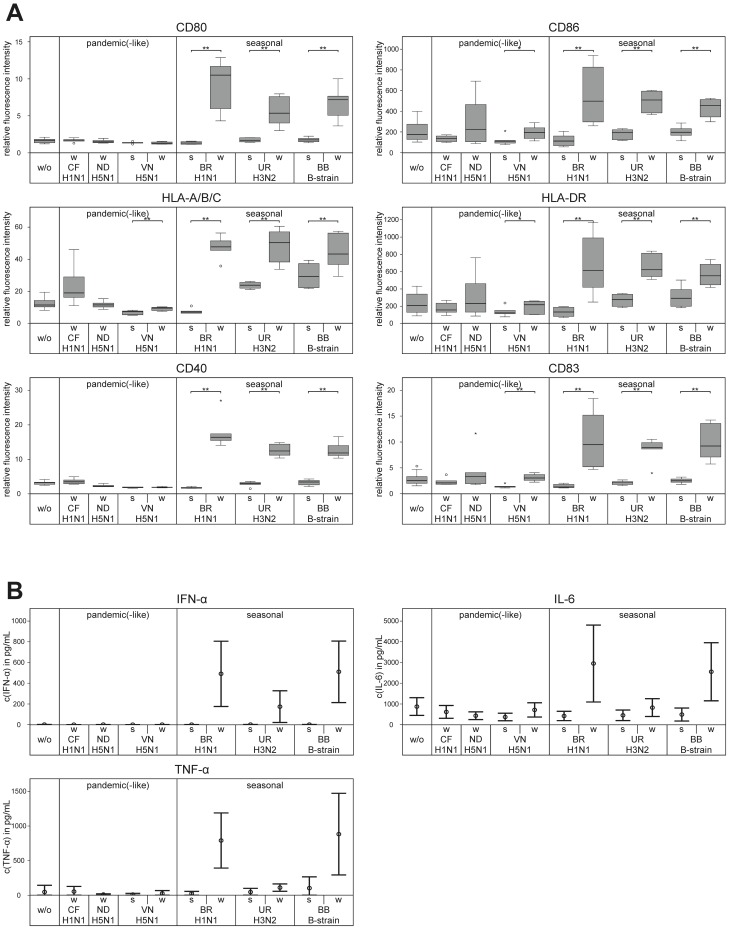
Split virus preparation substantially decreases the ability to activate and mature DC. (A) Immature DC were incubated for 48 h with different pandemic (-like) and seasonal influenza virus vaccines at 10 µg/mL (referring to HA content) and were subsequently analyzed by flow cytometry for expression of maturation markers on viable 7AAD-negative cells. Representative data of a single donor are shown in Fig. 1. Relative fluorescence intensity was calculated for each individual marker from MFI value of marker-specific staining divided by MFI value of the related IgG isotype control staining and was measured in 6 randomly selected donors. Summarized data are shown here as box blot diagrams. Split virus formulations were unavailable from pandemic (-like) strains A/H1N1-California and A/H5N1-Indonesia. P-values comparing the maturation effect of whole virus vaccines and related split virus vaccines on DC were calculated using two-tailed paired Wilcoxon signed-rank test (*, p<0.10; **, p<0.05). (B) After incubation of immature DC for 48 h with different pandemic (-like) and seasonal influenza virus vaccines, culture supernatants of four randomly selected donors were measured for IFN-α by ELISA, as well as for IL-6 and TNF-α by cytometric bead array. Graphs show mean concentrations (± SD) derived from experiments in all four healthy donors. For abbreviations of vaccines see Table 1. w/o, without.

Whole virus vaccines were analyzed for the ability to induce maturation in monocyte-derived immature DC of healthy individuals. Vaccines were available from seasonal strains A/H1N1-Brisbane, A/H3N2-Uruguay, and B/Brisbane, as well as from recent pandemic-like avian and pandemic swine-origin strains A/H5N1-Indonesia, A/H5N1-Vietnam, and A/H1N1-California, respectively (Table 1). Flow cytometry data on DC showed that incubation with seasonal whole virus vaccines strongly increased the expression of CD80, CD86, HLA-A/B/C, HLA-DR, CD40, CD83, consistent with a phenotype of mature DC (Fig. 1, Fig. 2A). This up-regulation was absent or observed at much lower level if pandemic (-like) whole virus vaccines were added to DC.

Supernatants of DC cultures were measured for IFN-α, IL-6, and TNF-α (Fig. 2B) usually secreted by DC during the maturation process. Whereas whole virus vaccines of seasonal strains A/H1N1-Brisbane and B/Brisbane strongly induced the production of IFN-α, IL-6, and TNF-α, the whole virus vaccine of seasonal strain A/H3N2-Uruguay increased cytokine production to a lower extent. Additionally, pandemic (-like) whole virus vaccines were largely unable to trigger secretion of IFN-α, IL-6, and TNF-α in DC. None of the whole virus vaccines stimulated DC to produce significant levels of IL-12 p70 (data not shown).

### Stronger DC maturation by whole virus vaccines compared to split virus counterparts

Split virus vaccines that had been prepared from the same individual virus stocks as whole virus vaccines were also analyzed for impact on DC phenotype by flow cytometry. The direct comparison with whole virus vaccines consistently showed that split virus preparations had a much lower ability to up-regulate the expression of DC maturation markers (Fig. 2A). In addition, split virus formats did not activate DC to secrete IFN-α, IL-6, TNF-α (Fig. 2B), and IL-12 p70 (data not shown).

### Pre-existing T-cell immunity to pandemic (-like) and seasonal influenza strains

T-cell reactivity was analyzed against the entire panel of pandemic (-like) and seasonal influenza vaccines in healthy volunteers. During *in vitro* pre-pulsing with vaccine preparations, DC received a maturation cytokine cocktail consisting of IL-6, TNF-α, IL-1β, and PGE_2_ to ensure optimal APC function. This appeared necessary, because the preceding experiments had shown a lower DC maturation ability of pandemic (-like) versus seasonal vaccines as well as of split virus versus whole virus preparations. However, DC maturation cytokines were not essentially required for recognition, as they generated only a slight albeit non-significant increase of vaccine-reactive CD4^+^ and CD8^+^ T cells in IFN-γ ELISpot assay (Fig. S1).

**Figure 3:**
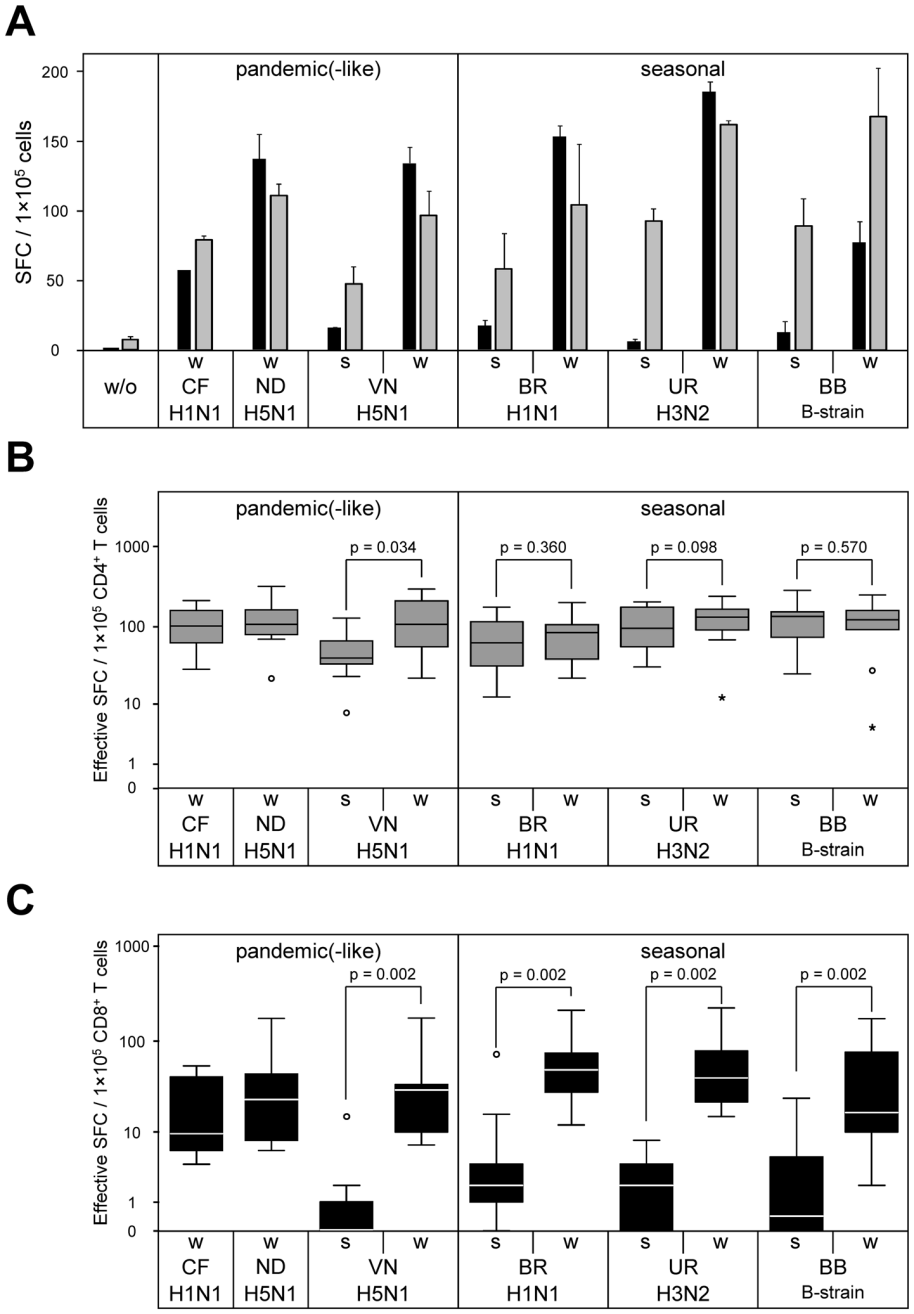
CD8^+^ T-cell reactivity to whole virus is superior compared to split virus preparations. CD4^+^ and CD8^+^ T cells purified from PBMC of healthy individuals were screened for IFN-γ ELISpot reactivity to autologous DC pre-loaded with 10 µg/mL of influenza whole virus and related split virus vaccine formulations. DC also received maturation cytokines during vaccine pulsing. (A) Representative data obtained from donor HD20 with 1×10^5^ CD4^+^ T cells (grey columns) or 1×10^5^ CD8^+^ T cells (black columns) plated per well are shown. (B, C) Reactivity to influenza whole virus and related split virus vaccines were measured in 10 randomly selected healthy individuals as described in (A). Box plot diagrams include IFN-γ ELISpot data from purified CD4^+^ (B) and CD8^+^ (C) T cells. Effective SFC were determined by subtraction of background spot numbers (w/o vaccine) from spot numbers induced by each individual vaccine. *P*-values were calculated by two-tailed paired-sample Wilcoxon signed-rank test.

Vaccine-loaded matured DC were subsequently used as APC to stimulate autologous CD4^+^ and CD8^+^ T cells of 10 healthy individuals in IFN-γ ELISpot assay. As shown in detail for a single representative donor, IFN-γ secreting CD4^+^ and CD8^+^ T cells were observed upon stimulation with every influenza whole virus vaccine preparation tested (Fig. 3A). Data summarized from the entire study cohort demonstrated that the frequencies of whole virus vaccine reactive CD4^+^ precursors were highest for seasonal strains A/H3N2-Uruguay and B/Brisbane, and were slightly lower for seasonal strain A/H1N1-Brisbane and all pandemic (-like) strains (Fig. 3B). Median numbers (and ranges) per 10^5^ CD4^+^ T cells were 105 (30–217), 110 (23–324), 112 (23–300), 87 (23–204), 135 (13–245), and 126 (5–255) for swine-origin A/H1N1-California, avian A/H5N1-Indonesia, avian A/H5N1-Vietnam, A/H1N1-Brisbane, A/H3N2-Uruguay, and B/Brisbane, respectively. In contrast, frequencies of whole virus vaccine reactive CD8^+^ T cells were approximately 2- to 10-fold lower than that of CD4^+^ counterparts, as determined in the same donor cohort (Fig. 3C). Highest immunogenicity for CD8^+^ T cells was observed with seasonal vaccines A/H1N1-Brisbane and A/H3N2-Uruguay, whereas CD8^+^ responses to seasonal vaccine B/Brisbane and all pandemic (-like) vaccines were of lower magnitude. Median values (and ranges) per 1×10^5^ CD8^+^ T cells were 10 (5–54), 24 (6–173), 30 (7–173), 50 (13–211), 40 (15–223), and 18 (2–170) for A/H1N1-California, A/H5N1-Indonesia, A/H5N1-Vietnam, A/H1N1-Brisbane, A/H3N2-Uruguay, and B/Brisbane, respectively.

### Stronger CD8^+^ T-cell responses to whole virus versus split virus vaccines

In the same donor cohort (n = 10), IFN-γ ELISpot reactivity of CD4^+^ T cells to whole virus and split virus vaccine preparations was very similar for most influenza strains (Fig. 3B). In contrast, superior immunogenicity of whole virus compared to split virus vaccines was much more consistent in CD8^+^ T cells, where it was detectable with strong statistical significance for all tested seasonal and pandemic (-like) strains (Fig. 3C). In direct comparison, whole virus preparations stimulated a median of 27 (18–60)- fold more influenza-specific CD8^+^ T-cell precursors compared to split virus counterparts. The data also showed that maturation of DC by exogenous cytokines (as performed in the described experiments) was completely unable to compensate for the poor effects of split virus vaccine preparations to stimulate influenza-specific memory CD8^+^ T cells *in vitro*.

The improved immunostimulatory capacity of influenza whole virus versus split virus vaccines to CD8^+^ T cells but not to CD4^+^ T cells was confirmed using flow cytometry staining for intracellular IFN-γ (Fig. S2). The influenza-reactive IFN-γ producing CD8^+^ T cells were observed in central memory (CD45RA^−^CCR7^+^), effector memory (CD45RA^+^CCR7^−^) and late effector (CD45RA^+^CCR7^−^) T-cell subsets, respectively (data not shown).

### Whole virus vaccines gain access to the cytosol of DC

Split virus vaccines prepared from whole virus vaccines by single Triton X-100 detergent incubation showed a similar distribution and concentration of proteins as whole virus formulations in SDS-PAGE analysis, but had lost structural integrity of virus particles according to electron microscopy pictures (Fig. S3). They had a much lower *in vitro* ability to mature DC and to stimulate IFN-γ-secreting memory CD8^+^ T cells compared to whole virus counterparts. In contrast, split virus and whole virus vaccines showed a similar efficiency in inducing IFN-γ secretion by pre-existing virus-specific CD4^+^ T cells. These data suggested that the structural integrity of whole virus vaccines is of crucial importance for eliciting antiviral CD8^+^ (but not CD4^+^) memory T-cell responses, but the exact mechanism behind this hypothesis was unclear. One possible explanation could be that whole virus vaccine production preserved the ability of virus material to fuse with endolysosomal membranes inside the DC. Subsequently, viral proteins could gain access to the cytosol, where they are degraded by proteasomal enzymes and enter the MHC class I pathway for successful stimulation of influenza-specific CD8^+^ T cells.

**Figure 4:**
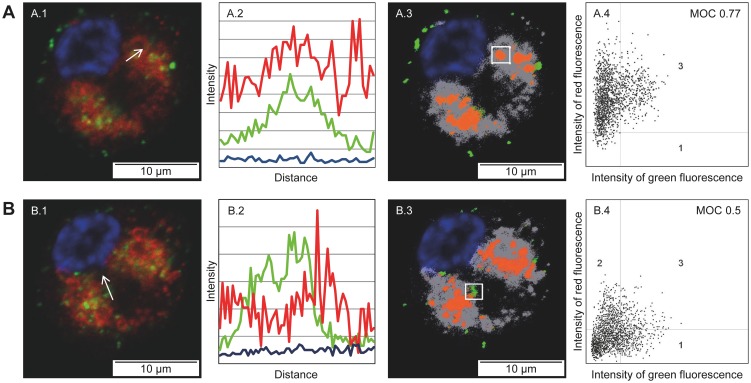
Whole virus vaccine material can be detected outside the endolysosomal compartment. Confocal LSM analysis was performed on immature DC after 4/H3N2-Uruguay whole virus vaccine. DC were pre-treated with the irreversible proteasome inhibitor epoxomicin for 4 h before vaccine was added. (A) and (B) show two different sectional planes of the same DC that was selected as a representative example of the entire sample. A.1/B.1 show LSM pictures, including nucleus (Hoechst 33342) marked in blue color, endolysosomal compartment (Lamp1/Rab5) in red color, and nucleoprotein (NP) of H3N2 in green color, respectively. (A.2/B.2) Graphs represent the fluorescence intensity of staining dyes used in A.1/B.1. White arrows in A.1/B.1 mark the measuring points from basis to arrowhead. (A.3/B.3) To further analyze the localization of NP within the DC, A.1/B.1 pictures were post-processed. Blue color means Hoechst positive only, grey color means Lamp1/Rab5 positive only, orange color means Lamp1/Rab5 as well as NP positive, green color means NP positive only. Thresholds are indicated in A.4/B.4. (A.4/B.4) Diagrams show the intensities of red (Lamp1/Rab5) and green (NP) fluorescence within white rectangles of A.3/B.3. To quantify colocalization of NP and Lamp1/Rab5 within the white rectangle of A.3/B.3 Mander's overlap coefficient (MOC) was calculated. Value range 0–1 (0: no colocalization, 1: all pixels colocalize).

To investigate this hypothesis confocal laser scanning microscopy (LSM) was performed in order to localize influenza virus material inside DC. After loading with whole virus vaccine of the seasonal A/H3N2-Uruguay strain, viral nucleoprotein (NP) was clearly observed at the outer cell membrane of DC as well as intracellularly (Fig. 4). Post-processing data analysis showed that most intracellular NP co-localized with the endolysosomal compartment (Fig. 4A). However, in several DC intracellular NP signals could also be detected separate from endolysosomal structures, suggesting that whole virus material had escaped the endolysosome (Fig. 4B). In contrast, influenza NP was not detected inside or outside DC after loading them with the split virus A/H3N2-Uruguay vaccine (Fig. S4). This suggested that the epitope of the used NP antibody had been most likely affected by the splitting process during split virus vaccine production.

**Figure 5:**
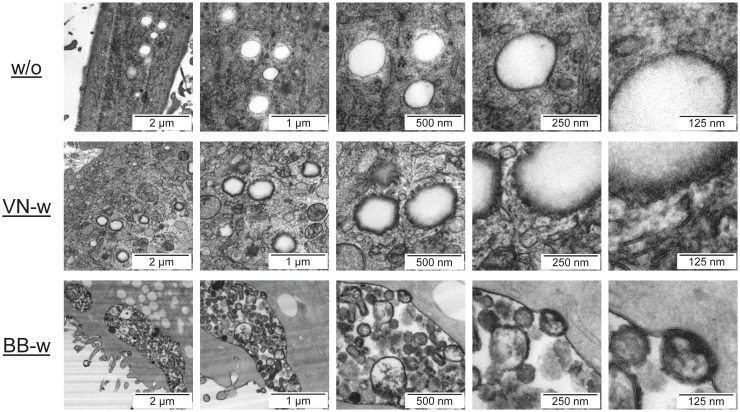
Endolysosomal escape of whole virus vaccine material. Immature DC were incubated for 2(w/o) or with whole virus vaccines of pandemic-like avian strain A/H5N1-Vietnam (VNw) or seasonal strain B/Brisbane (BBw). Subsequently, samples were processed and analyzed by TEM using a Zeiss 912 Omega microscope operated at 120 kV. Pictures were taken at 5 increasing magnifications to allow better detection and tracking of virus material.

Vaccine-loaded DC were also analyzed by TEM. The pandemic-like avian A/H5N1-Vietnam whole virus vaccine consisted of filamentous particles that located at the periphery of endolysosomal structures (Fig. 5). At highest magnification these elongated particles could even be detected close to the endolysosomal compartment in the cytosol (Fig. 5 and Fig. S5). In contrast, seasonal B/Brisbane (Fig. 5) and A/H3N2-Uruguay (not shown) whole virus vaccines formed round-shaped and oval particles of variable size, which could be easily located inside huge endolysosomal vesicles. Some of them formed distinct endolysosomal membrane bulges. This observation might describe an ongoing membrane fusion process, which however is difficult to demonstrate with sufficient certainty by static pictures. Similar vaccine-related structures were not found in DC samples that were pre-pulsed with TBS buffer (Fig. 5) or split virus vaccines (data not shown). Split-virus preparations consisted of disintegrated small fragments of variable dimensions and forms (exemplarily shown in Fig. S3C), making it impossible to distinguish them from intracellular structures of DC.

**Figure 6:**
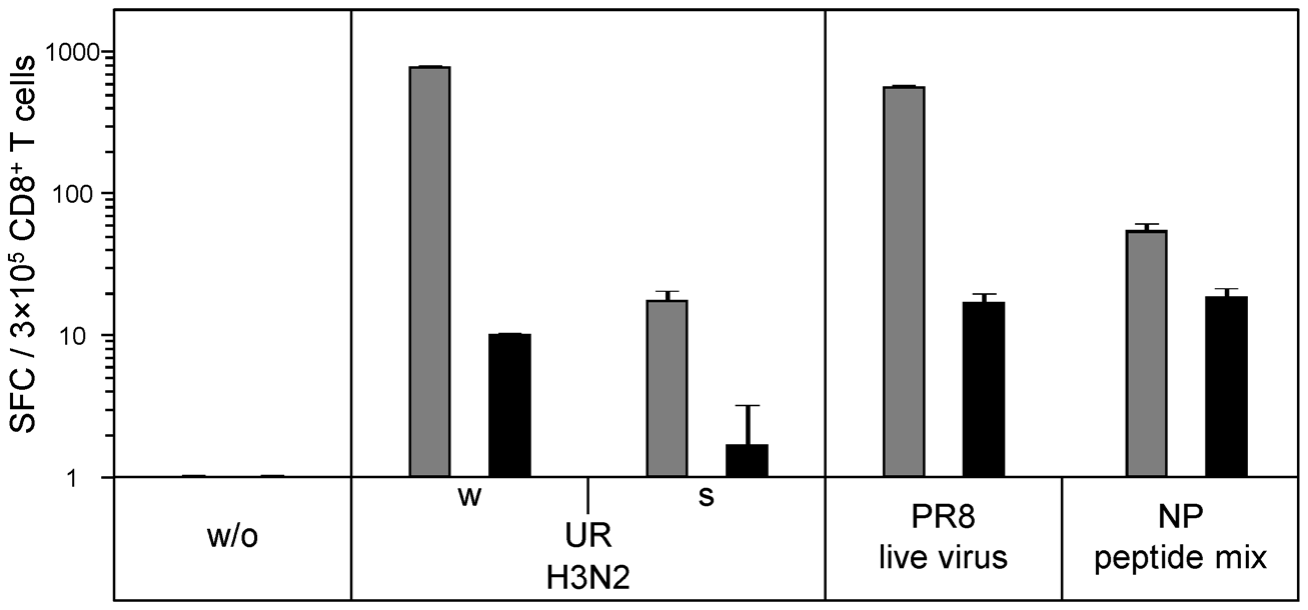
Proteasome inhibition strongly reduces CD8^+^ T-cell reactivity to whole virus vaccine. CD8^+^ T cells purified from PBMC of healthy donor HD21 were screened for IFN-γ ELISpot reactivity to autologous immature DC loaded with seasonal A/H3N2-Uruguay whole virus or split virus vaccines, or an influenza A/H3N2 nucleoprotein oligopeptide mix. Targets also included DC infected with live influenza virus of the Puerto Rico 8 lab strain. During loading or infection, respectively, DC were treated with epoxomicin (black columns) or DMSO solvent (grey columns), which both did not reduce cell viability in trypan blue staining. Data are representative of 3 experiments performed in different donors.

To obtain additional evidence that whole virus vaccine material enters the cytosolic compartment of DC, proteasomes were irreversibly inhibited by epoxomicin before loading cells with the whole virus A/H3N2-Uruguay vaccine. DC infected with live influenza virus of the Puerto Rico 8 laboratory strain was included as positive control. Figure 6 shows that short-time epoxomicin pre-treatment strongly reduced CD8^+^ T-cell reactivity to whole virus A/H3N2-Uruguay vaccine and live Puerto Rico 8 virus by 99% and 97%, respectively. Epoxomicin-induced inhibition was also observed at significant levels, when CD8^+^ T-cell reactivity was measured to the split virus A/H3N2-Uruguay vaccine (i.e. 90%) as well as to an oligopeptide mix derived from the influenza A/H3N2 nucleoprotein (i.e. 66%). As epoxomicin pre-treatment did not decrease viability of DC according to trypan blue staining controls, nearly complete loss of CD8^+^ T-cell reactivity to whole virus vaccine in proteasome-inhibited DC argued against a mere toxic effect but suggested that whole virus vaccine material effectively enters the cytosol for proteasomal digestion and processing by the MHC class I pathway.

## Discussion

The study shows that whole virus influenza vaccines prepared from major seasonal A and B strains have a strong *in vitro* ability to mature human monocyte-derived DC. Thus whole virus vaccine production including double-inactivation by formalin and ultraviolet treatment can preserve important pathogen-associated molecular patterns (PAMP) of seasonal influenza viruses for binding pattern recognition receptors (PRR) to induce DC maturation [20]. However, the magnitude of this response can significantly differ among seasonal vaccines, as observed for the reduced cytokine production upon incubation with the A/H3N2-Uruguay preparation. Secretion of IL-12 p70 was generally not found, neither by maturing DC with a cytokine cocktail [17] nor by any of the used whole virus vaccines. This observation might relate to the experimental design, in which pure monocyte-derived DC were unable to interact with T cells and NK cells. Both cell types were reported to effectively trigger IL-12 p70 production in DC [21].

Pandemic (-like) swine-origin and avian whole virus vaccines had an overall lower ability to induce DC maturation. This observation was somewhat surprising because the major immune response triggering receptors TLR 7 and TLR 8 expressed by DC are known to bind a broad pattern of viral single-stranded RNA (ssRNA) [22], [23], and should therefore not distinguish between RNA of seasonal and pandemic (-like) strains. Additionally, the deficiency of pandemic-like H5N1 whole virus vaccines to induce secretion of inflammatory cytokines (i.e. IL-6, TNF-α, and IFN-α) was unexpected, since the infection of humans with live H5N1 viruses was reported to be associated with hypercytokinemia [24]. A potential explanation might be a different matrix of the seasonal whole virus preparations compared to that of the pandemic (-like) whole virus counterparts as the latter contained the excipient polysorbate 80 to ensure homogeneity during storage at +4°C [14]. This additive could have changed the physical structure of biologically relevant vaccine components including PAMP.

In contrast to whole virus vaccines, split virus counterparts produced by single Triton X-100 treatment completely lost the ability to induce DC maturation in all tested seasonal and pandemic-like strains. Most likely reasons for this observation are the detergent-induced structural disintegration and the loss of functionally active RNA components of influenza viruses (Fig. S3) [25]. It has been reported that DC use several different PRR to bind virus-specific ssRNA [26]. First, TLR 7 and TLR 8, which both are exclusively expressed in the endolysosomal membrane, acquire ssRNA that is released from influenza virions during the acidification and maturation process in the endosome [22], [23]. Because of preserved membrane fusion activity, only whole virus vaccines appear able to effectively deliver virus material including ssRNA into the cytosol. Here ssRNA could bind to cytosolic PRR, such as the retinoid acid inducible gene I (RIG-I) [27]. Consequently, due to the activation of both, endosomal as well as cytosolic PRR, whole virus vaccines appear superior to split virus vaccines in terms of the ability to activate and mature DC.

A most striking observation was that influenza whole virus vaccines pulsed on DC effectively stimulated IFN-γ secreting memory CD8^+^ T cells of every study participant *in vitro*. Median CD8^+^ T-cell reactivity was highest to both seasonal influenza A vaccines, and was slightly lower to the seasonal influenza B as well as to the pandemic (-like) avian and swine-origin influenza A vaccines. This difference in pre-existing immunity level of CD8^+^ T cells could be caused by the exposure history of donors to individual influenza virus strains. Notably, the immune-stimulatory effect on CD8^+^ T cells was also found if DC did not receive exogenous maturation cytokines during vaccine loading and if the pandemic (-like) whole virus vaccines with the observed deficiency to promote DC maturation were used. This observation clearly demonstrates that whole virus vaccine particles itself do not need to possess significant DC maturation capability in order to stimulate IFN-γ production in virus-specific CD8^+^ T cells. In contrast, split virus vaccine counterparts showed a considerably lower (>1-log) ability to activate virus-specific memory CD8^+^ T cells. The results suggested that whole virus vaccine material effectively enters the cytosol after uptake into DC, where influenza proteins are digested by the proteasome complex to fragments that move into the MHC class I pathway [28]. To confirm this hypothesis DC received short pre-treatment with the irreversible proteasome inhibitor epoxomicin during vaccine loading, which resulted in an overall decrease of influenza-specific CD8^+^ T-cell reactivity. Because DC viability was not impaired directly after epoxomicin treatment, reduced immune-stimulatory activity might be explained by other reasons, e.g. drug-induced NF-κB inactivation or delayed toxicity in DC [29]. However, the epoxomicin effect was clearly most pronounced in DC loaded with whole virus vaccine or live virus, particularly compared to DC pulsed with overlapping nucleoprotein oligopeptides that showed consistent residual ability to activate virus-specific CD8^+^ T cells. Altogether this suggested that the production of CD8^+^ T-cell epitopes from whole virus vaccine material essentially requires proteasome function, similarly to what is known for live virus [30]. In addition to LSM and TEM data, it supports the hypothesis that whole virus vaccine particles effectively enter the cytosol for proteasomal processing and presentation by the MHC class I pathway. Although the level of CD8^+^ T-cell reactivity to split virus vaccines was found to be much lower compared to that against whole virus vaccines, it was also sensitive to epoxomicin treatment at reduced but still significant degree. These data suggest that split virus material may also escape the endolysosome and rely on proteosomal degradation. As an alternative explanation, epoxomicin may not only block the proteasome complex but also impact other processes involved in MHC class I presentation of split virus material.

There are contradictory reports whether inactivation of live influenza virus by heat and formalin abrogates the membrane fusion activity and other important features that can influence the immunogenicity for CD8^+^ T cells [11], [31]. Apart from clear methodical differences, the vaccine production process used in previous studies did not achieve total virus inactivation with a large safety margin, as obtained in the current work using a stringent double-inactivation procedure for whole virus vaccines that has been approved by major regulatory authorities [13]. Most importantly, earlier studies could not definitely exclude if low amounts of residual functionally active virus had infected recipient cells, thereby stimulating CD8^+^ T cells by the natural route. In contrast, our data show that influenza NP derived from a double-inactivated whole virus vaccine could clearly be detected inside the cells but outside the endolysosomal compartment by confocal LSM. Additionally, TEM analysis providing much higher resolution suggested ongoing fusion of elongated and round-shaped whole virus vaccine particles with endolysosomal membranes and localization of filamentous particles in the cytosol in immediate proximity to endolysosomes. In summary, confocal LSM and TEM data suggest that a fraction of whole virus vaccine particles is indeed capable of escaping endolysosomal compartments. However, as it was not possible to visualize the structurally disintegrated components of split virus vaccines inside DC using confocal LSM or TEM, these data do not allow a direct comparison of whole and split virus vaccines.

The *in vitro* immune response of memory CD4^+^ T cells against seasonal and pandemic (-like) influenza vaccines showed major differences compared to that of the CD8^+^ subset. First, the level of reactivity was approximately 2- to 10-fold higher in CD4^+^ versus CD8^+^ T cells and did not vary considerably between seasonal and pandemic (-like) strains. In addition, split virus and whole virus vaccines were nearly equally efficient in stimulating virus-specific memory CD4^+^ T cells, indicating that split virus material gained sufficient access to the endolysosomal MHC class II pathway [32]. However, inefficient DC maturation as demonstrated herein for split virus vaccines might significantly reduce the overall magnitude and persistence of the antiviral T-cell response [33].

The study confirms previous reports showing that heterosubtypic CD4^+^ and CD8^+^ T-cell reactivity to seasonal and pandemic (-like) influenza viruses is common in the general human population [34]–[37]. Several investigators have also demonstrated that a major part of the influenza-specific memory T-cell response in healthy individuals is directed against peptide epitopes shared between different seasonal and pandemic (-like) strains [4], [35], [38], [39]. In this regard IFN-γ spot numbers exceeding the level of reactivity to pandemic-like H5N1 strains most likely represent recognition of epitopes encoded specifically by seasonal strains. It remains open at this point whether memory T cells cross-reacting against conserved epitopes of major influenza strains, which are frequently reactivated by virus exposure during influenza seasons, are sufficient to provide some level of protection against newly emerging pandemic virus strains [39], [40].

In summary, seasonal and pandemic (-like) influenza whole virus vaccines loaded on monocyte-derived DC effectively stimulated heterosubtypic virus-specific CD4^+^ as well as CD8^+^ memory T cells *in vitro*. The (re)-activation of virus-specific CD8^+^ T cells did not depend on DC with fully mature phenotype, as observed for the pandemic (-like) strain vaccines. Directly compared split virus vaccines were much less potent in stimulating CD8^+^ T cells, either due to abrogated membrane fusion ability or inability to activate DC for DC-intrinsic mechanisms of cross-presentation. As most CD8^+^ CTL are directed against conserved internal influenza virus proteins [41], stimulation of CD8^+^ responses is of major importance to establish broad cross-protection against seasonal as well as new pandemic (-like) strains. In this regard the study provides a clear rationale to use whole virus vaccines instead of split virus counterparts, if *in vivo* stimulation of coordinate CD4^+^ as well as CD8^+^ T-cell immunity against influenza viruses represents a main aim of vaccination. Prospective clinical trials directly comparing split virus and whole virus vaccines are required to find out if the presented *in vitro* data can be confirmed *in vivo*. Such trials should include a careful monitoring of the full repertoire of influenza-specific CD4^+^ and CD8^+^ T cells, for which the current data are certainly helpful. The presumably improved immunogenicity of influenza whole virus vaccines would then be calculated on the risk of vaccine-induced side effects, which has been reported by some investigators to be higher than that of split virus and subunit seasonal influenza vaccines [42]–[44].

## Supporting Information

Figure S1 **Treatment of vaccine-loaded DC with maturation cytokines can improve detection of H5N1 influenza-specific T cells.** CD4^+^ and CD8^+^ T cells purified from PBMC of 5 randomly selected healthy donors were analyzed for reactivity to the avian A/H5N1-Vietnam whole virus vaccine in IFN-γ ELISpot assay. APC were autologous DC that were pre-incubated for 48 h with 10 µg/mL vaccine preparation. During antigen pulsing, DC were either treated with maturation cytokines IL-6, TNF-α, IL-1β, and PGE_2_ (‘cocktail’) or were left untreated (w/o ‘cocktail’). (A) Data obtained in healthy individual HD26 with 1×10^5^ CD8^+^ T cells (black columns) or 1×10^5^ CD4^+^ T cells (grey columns) plated per well. (B, C) Box plot diagrams include data of CD8^+^ (B) and CD4^+^ (C) responses in 5 healthy volunteers. *P*-values were calculated using the two-tailed paired-sample Wilcoxon signed-rank test. SFC, spot-forming cells; w/o, without.
(TIF)

Figure S2 **Whole virus vaccines induce stronger CD8^+^ but similar or lower CD4^+^ T cell responses in comparison to their split virus counterparts.** IFN-γ production of CD8^+^ and CD4^+^ T cells purified from PBMC of healthy individuals was measured by intracellular cytokine staining and ELISpot after stimulation with autologous monocyte-derived DC pre-loaded with influenza whole virus and split virus vaccine formulations. DC had been treated with maturation cytokines during pulsing with seasonal A/H3N2-Uruguay or A/H1N1-Brisbane vaccines. (A) In intracellular cytokine staining, cells were gated on CD3^+^CD4^+^ (or CD3^+^CD8^+^) and analyzed for intracellular IFN-γ. Fractions (%) of IFN-γ positive CD8^+^ or CD4^+^ T cells are added. (B) IFN-γ ELISpot data obtained from the same donor with 1×10^5^ CD4^+^ T cells (grey columns) or 1×10^5^ CD8^+^ T cells (black columns) plated per well are shown.
(TIF)

Figure S3 **Protein composition, RNA content, and physical structure of whole virus and split virus vaccines.** Shown data are from vaccine formulations of seasonal strain B/Brisbane (BB). (A) Semiquantitative SDS-PAGE analysis indicated similar distribution and concentration of proteins in split virus (s) and whole virus (w) preparations. (B) Electropherograms showed clearly different size distribution of RNA from split virus versus whole virus preparations. RNA of split virus preparation was considerably more degraded and of much smaller size. Split virus and whole virus preparations contained 7 pg and 627 pg RNA per µL per dose (500 µL), respectively (not shown). (C) TEM analysis of vaccine samples using a FEI Tecnai 10 microscope operated at 100 kV. Pictures were taken with an Olympus Veleta camera. The size scale is indicated. FU, fluorescence units; nt, nucleotides; kDa, kiloDalton.
(TIF)

Figure S4 **Confocal LSM analysis of DC loaded with whole and split virus vaccines.** Immature DC were analyzed after 4 h incubation with seasonal A/H3N2-Uruguay whole virus vaccine (URw), its corresponding split virus vaccine (URs), or PBS as negative control. The nucleus (Hoechst 33342) is stained in blue color, whereas endolysosomal compartments (Lamp1/Rab5) are marked in red color, and nucleoprotein (NP) of H3N2 in green color, respectively.
(TIF)

Figure S5 **Particles from avian A/H5N1-Vietnam whole virus vaccine can be detected in the cytosol.** Similar to Figure 4, immature DC were incubated with whole virus vaccine of avian strain A/H5N1-Vietnam (VNw) for 2 h, processed and analyzed by TEM. Elongate particles of the whole virus vaccine presumably escaping the endolysosomal compartment, as well as particles localized within the cytosol can be observed. The white arrow in the upper left picture demonstrates the localization of the focused endolysosome within the DC.
(TIF)
