# Supplementary figures and images for: Superior In Vitro Stimulation of Human CD8+ T-Cells by Whole Virus versus Split Virus Influenza Vaccines
Source: PLoS One. 2014 Jul 29;9(7):e103392. doi: 10.1371/journal.pone.0103392 (PMC4114834; doi:10.1371/journal.pone.0103392)

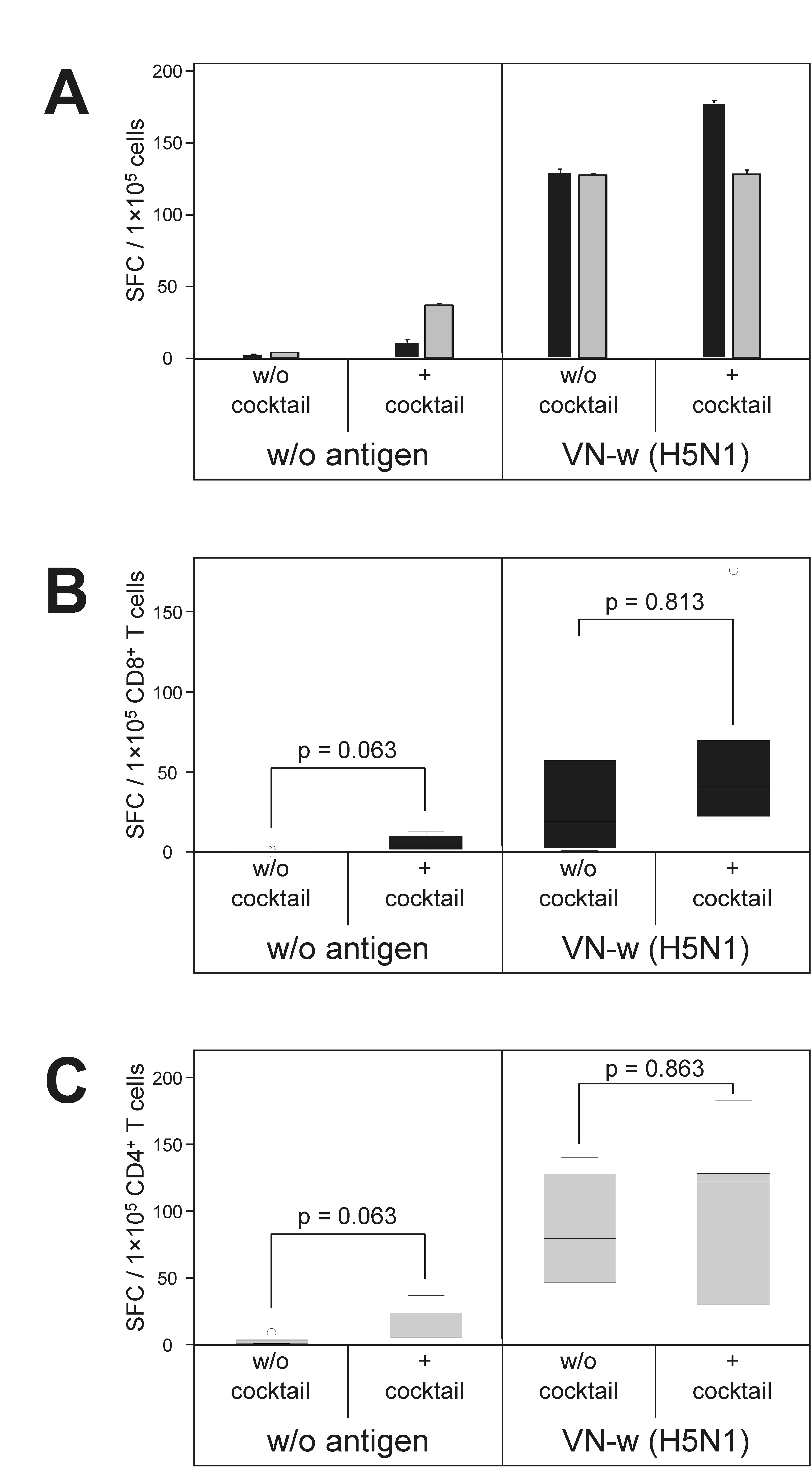

Supplement: Figure S1 — Treatment of vaccine-loaded DC with maturation cytokines can improve detection of H5N1 influenza-specific T cells. CD4+ and CD8+ T cells purified from PBMC of 5 randomly selected healthy donors were analyzed for reactivity to the avian A/H5N1-Vietnam whole virus vaccine in IFN-γ ELISpot assay. APC were autologous DC that were pre-incubated for 48 h with 10 µg/mL vaccine preparation. During antigen pulsing, DC were either treated with maturation cytokines IL-6, TNF-α, IL-1β, and PGE2 (‘cocktail’) or were left untreated (w/o ‘cocktail’). (A) Data obtained in healthy individual HD26 with 1×10^5^ CD8+ T cells (black columns) or 1×10^5^ CD4+ T cells (grey columns) plated per well. (B, C) Box plot diagrams include data of CD8+ (B) and CD4+ (C) responses in 5 healthy volunteers. P-values were calculated using the two-tailed paired-sample Wilcoxon signed-rank test. SFC, spot-forming cells; w/o, without. (TIF) [file pone.0103392.s001.tif]

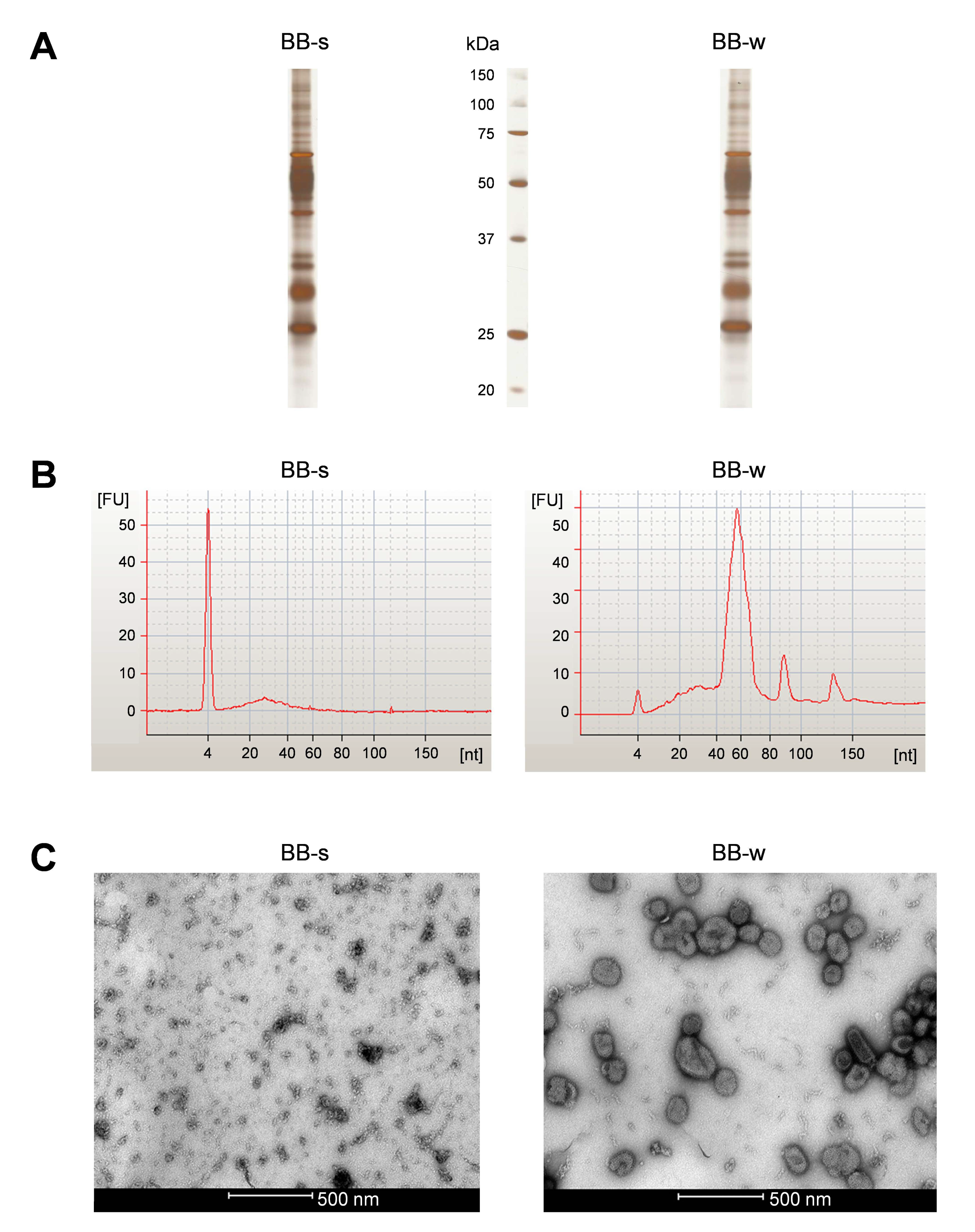

Supplement: Figure S3 — Protein composition, RNA content, and physical structure of whole virus and split virus vaccines. Shown data are from vaccine formulations of seasonal strain B/Brisbane (BB). (A) Semiquantitative SDS-PAGE analysis indicated similar distribution and concentration of proteins in split virus (s) and whole virus (w) preparations. (B) Electropherograms showed clearly different size distribution of RNA from split virus versus whole virus preparations. RNA of split virus preparation was considerably more degraded and of much smaller size. Split virus and whole virus preparations contained 7 pg and 627 pg RNA per µL per dose (500 µL), respectively (not shown). (C) TEM analysis of vaccine samples using a FEI Tecnai 10 microscope operated at 100 kV. Pictures were taken with an Olympus Veleta camera. The size scale is indicated. FU, fluorescence units; nt, nucleotides; kDa, kiloDalton. (TIF) [file pone.0103392.s003.tif]

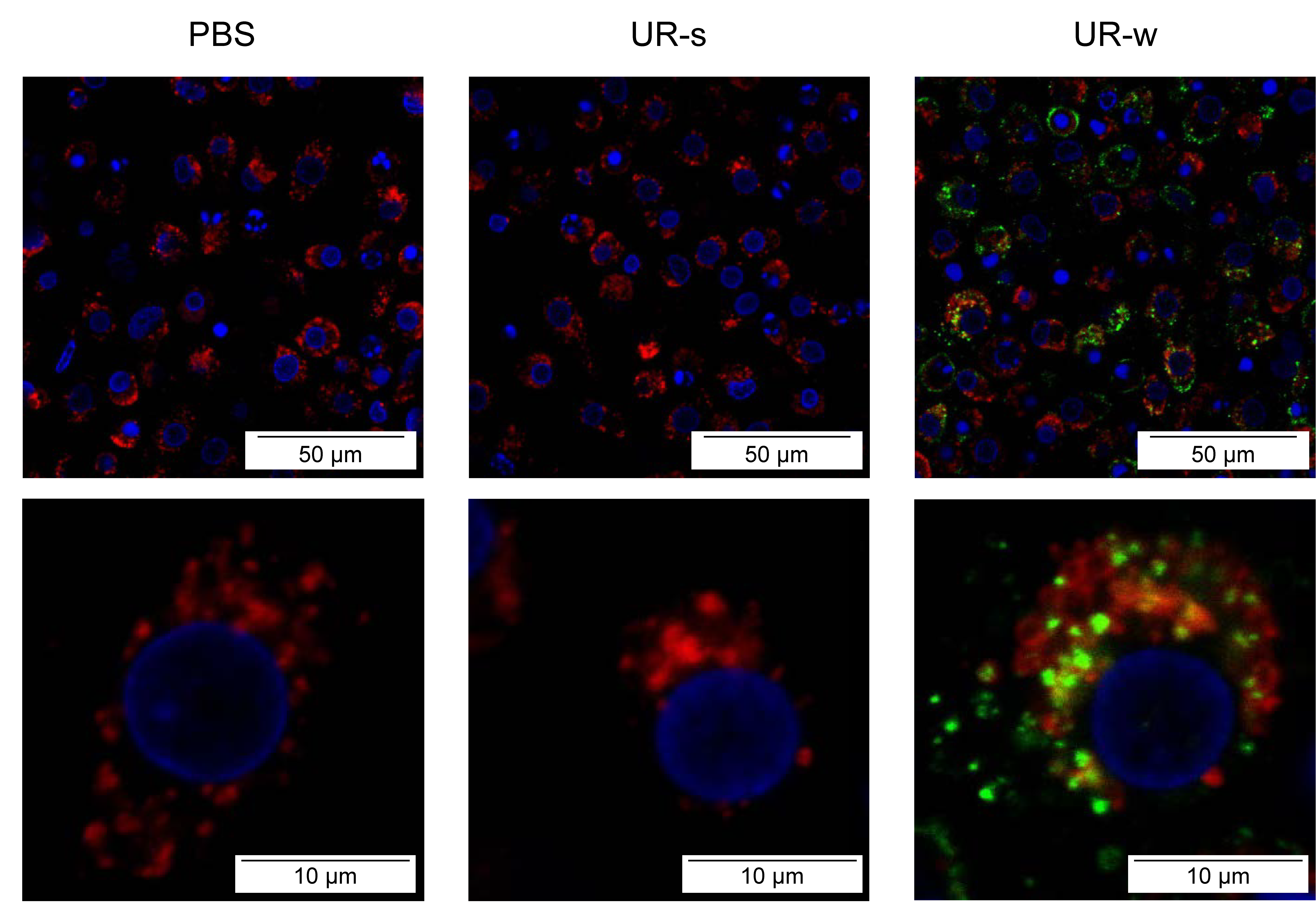

Supplement: Figure S4 — Confocal LSM analysis of DC loaded with whole and split virus vaccines. Immature DC were analyzed after 4 h incubation with seasonal A/H3N2-Uruguay whole virus vaccine (URw), its corresponding split virus vaccine (URs), or PBS as negative control. The nucleus (Hoechst 33342) is stained in blue color, whereas endolysosomal compartments (Lamp1/Rab5) are marked in red color, and nucleoprotein (NP) of H3N2 in green color, respectively. (TIF) [file pone.0103392.s004.tif]
